# Exploring the role of teacher empathy in student mental health outcomes: a comparative SEM approach to understanding the complexities of emotional support in educational settings

**DOI:** 10.3389/fpsyg.2025.1503258

**Published:** 2025-03-20

**Authors:** Joshua Ampofo, Geoffrey Bentum-Micah, Qian Xusheng, Binghai Sun, Rita Mensah Asumang

**Affiliations:** ^1^College of Education, Zhejiang Normal University, Jinhua, China; ^2^School of Psychology, Zhejiang Normal University, Jinhua, China; ^3^School of Teacher and Education, Shaoxing University, Shaoxing, China

**Keywords:** teacher empathy, student mental health outcomes, emotional support, student engagement, structural equation modeling

## Abstract

**Introduction:**

This study investigates the role of teacher empathy in influencing student mental health outcomes through a comparative Structural Equation Modeling (SEM) approach. Given the rising prevalence of mental health challenges among students, understanding the impact of empathetic teacher-student relationships is crucial.

**Methods:**

Utilizing a sample of 300 students from diverse educational settings, the research examines how teacher empathy affects student engagement and mental health. The study employs SEM to analyze the relationships between perceived teacher empathy, student engagement, and mental health outcomes.

**Results:**

Findings reveal that higher levels of perceived teacher empathy correlate with reduced stress, anxiety, and depression while enhancing student engagement in learning activities. Furthermore, student engagement mediates the relationship between teacher empathy and mental health outcomes.

**Discussion:**

The results underscore the necessity of fostering empathetic relationships within educational contexts to promote student resilience and well-being. Implications for teacher training programs and academic practices are discussed, emphasizing the importance of empathy in creating supportive learning environments that enhance student mental health and engagement.

## Introduction

The increasing prevalence of mental health challenges among students has prompted a critical examination of the factors that influence their psychological well-being. Among these factors, teacher empathy-the ability of educators to understand and resonate with their students’ emotional experiences has emerged as a vital component in fostering positive mental health outcomes. Research reveals that supportive teacher-student relationships are significantly associated with reduced levels of stress, anxiety, and depression among students (He et al., 2019). Moreover, students who regard their professors as empathic demonstrate higher levels of resilience, self-esteem, and overall life satisfaction (Wang, 2023).

Despite teacher empathy’s recognized importance, the complexities surrounding its impact on student mental health outcomes remain underexplored. Existing literature primarily focuses on the direct benefits of empathetic interactions; however, there is a pressing need to investigate how these dynamics operate across different educational contexts and how they may mediate or moderate various psychosocial outcomes (Guldeste et al., 2024). For instance, while some studies suggest that teacher empathy can lead to improved academic engagement and emotional support (Kianinezhad, 2023) it is crucial to understand the mechanisms that enable these relationships and how they vary by student population. This work intends to overcome these gaps by adopting a comparative Structural Equation Modeling (SEM) technique to evaluate the complicated links between teacher empathy, emotional support, and student mental health outcomes across multiple educational environments. By studying these variables through SEM, we can find potential mediating factors that promote or hinder the effectiveness of teacher empathy in fostering favorable mental health outcomes. This research seeks to advance theoretical understanding and inform practical applications that can enhance teacher training programs and foster empathetic practices within educational institutions. In summary, this investigation will explore the role of teacher empathy as a critical determinant of student mental health outcomes. By elucidating the complexities of emotional support within educational settings, this study aims to provide valuable insights into fostering empathetic relationships in schools to promote student resilience and psychological well-being.

## Theoretical framework

### Teacher empathy and mental health

The concept of teacher empathy has gained significant traction in educational research, particularly concerning its implications for student mental health outcomes. Empathy, defined as the ability to comprehend and share the experiences of others, is a multidimensional term that includes both cognitive and affective elements (Neumann et al., 2015). In education, teacher empathy involves recognizing and responding to student’s emotional needs, which is crucial in fostering supportive classroom environments (Jennings and Min, 2023). This literature review aims to clarify the terms related to teacher empathy and mental health while exploring their interconnections within educational settings.

Teacher empathy can be categorized into two primary dimensions: cognitive empathy and affective empathy. Cognitive empathy relates to the ability to understand another person’s perspective and emotional state, while affective empathy entails sharing and responding to those emotions (Bezzina, 2022). Research indicates that instructors who demonstrate high levels of empathy are better suited to establish positive emotional climates in their classrooms, which can lead to greater student engagement and academic accomplishment (Aldrup et al., 2022). For instance, Williams (2010) emphasize that teachers with solid empathic abilities can effectively identify students’ emotional cues, enabling them to respond sensitively to their needs.

### Teacher empathy in mental health

The correlation between teacher empathy and student mental health outcomes is intricate and multifarious. Recent research has begun to explore the direct connection between teacher empathy and student engagement. For instance, studies by Aldrup et al. (2024) indicate that teachers who demonstrate high levels of empathy not only support students’ emotional needs but also foster greater engagement in classroom activities. This direct link is crucial as it highlights how empathetic teaching practices can enhance students’ active participation in learning. Studies have shown that empathetic teachers can significantly influence students’ psychological well-being by providing emotional support that mitigates stress and anxiety (Derakhshan, 2022). For example, research by Wang and Wang (2023) highlights that teacher care, an extension of empathy, can improve student well-being by addressing students’ psycho-emotional needs. Conversely, some studies suggest that high levels of empathy may also contribute to emotional exhaustion among teachers, potentially impacting their mental health (Schoeps et al., 2019). This duality suggests that while teacher empathy can serve as a protective factor for students’ mental health, it may also pose risks for teachers themselves if not managed effectively.

The significance of teacher empathy in educational settings cannot be overstated, particularly concerning its impact on student mental health. Empathy in education is not merely a personal trait but a critical pedagogical approach that can significantly affect students’ psychological well-being. Research consistently shows that students who perceive their teachers as empathetic report lower levels of anxiety, depression, and stress (He et al., 2019; Derakhshan, 2022). For example, Zhang et al. (2022) found that students with higher perceptions of teacher empathy experienced enhanced emotional regulation, contributing to improved mental health outcomes.

In addition to direct emotional support from teachers, other factors such as peer relationships and family dynamics play a crucial role in shaping student mental health. Furlong et al. (2020) emphasize that robust peer support systems can act as buffers against student stressors. Their findings indicate that when students feel connected to their peers and supported by their teachers, they are more likely to report positive mental health outcomes. Furthermore, Losada-Baltar et al. (2021) highlight the importance of family involvement in education, revealing that supportive family environments correlate positively with students’ emotional well-being.

### Teacher empathy as a determinant of emotional support

Emotional support from teachers is critical in promoting positive mental health outcomes among students. Research indicates that empathetic interactions between teachers and students foster a sense of belonging and security in the classroom (Ibarra, 2022). These supportive relationships are associated with various psychosocial benefits, including increased motivation, self-esteem, and student resilience (Aldrup, 2017). Furthermore, studies have demonstrated that teacher empathy can enhance the quality of teacher-student interactions, leading to improved academic performance and social adjustment (Sun et al., 2023). Because teachers’ empathy dramatically affects how well their students’ mental health does, teaching teachers how to be empathetic is essential. Developing empathic skills among educators can enhance their ability to provide emotional support effectively and manage classroom dynamics positively (Jennings and Greenberg, 2009). Furthermore, ongoing professional development focused on emotional intelligence may help mitigate the potential adverse effects of high empathic engagement on teachers’ mental health (Zoromba et al., 2023).

Emotional support from teachers is integral to fostering positive mental health outcomes among students. Research indicates that empathetic interactions between teachers and students create a sense of belonging and security within the classroom (Ibarra, 2022). This supportive relationship enhances students’ motivation and contributes to their overall resilience (Aldrup et al., 2017). For instance, Hen (2020) found that when teachers respond sensitively to students’ emotional cues, it significantly mitigates feelings of isolation or anxiety, promoting engagement.

Moreover, the quality of teacher-student relationships is pivotal in determining how effectively emotional support translates into improved mental health outcomes. Studies by Sun et al. (2023) demonstrate that high-quality teacher-student interactions characterized by empathy lead to better academic performance and social adjustment among students. These findings suggest that fostering strong relationships between teachers and students is essential for creating an environment conducive to learning and emotional growth.

### Student engagement in teacher empathy

Education research has been paying increasing attention to teacher empathy’s role in fostering student engagement, mainly in improving students’ mental health outcomes. It is possible to describe teacher empathy as the capacity of educators to comprehend and share their pupils’ sentiments and points of view. This capacity involves both the emotional and cognitive components of the student experience (Tettegah, 2007). This literature review examines the relationship between teacher empathy and student engagement, highlighting the mechanisms through which empathetic interactions can enhance students’ emotional and academic experiences. Teacher empathy involves recognizing and responding to students’ emotional needs and creating a supportive classroom environment that promotes engagement (Weisz et al., 2022). According to Rogers et al. (2013), when teachers perceive situations from their students’ perspectives, they significantly enhance the likelihood of effective learning. Students’ general involvement is greatly enhanced by this compassionate approach, which helps to create rapport and cultivates a sense of belonging (Zhang, 2022). Research repeatedly reveals a strong association between teacher empathy and student involvement across various educational situations. For instance, studies have shown that teachers who exhibit high levels of empathy can reduce students’ stress levels, enhancing their motivation and willingness to participate actively in classroom activities (Kianinezhad, 2023). Furthermore, empathic teachers are better equipped to create a positive emotional atmosphere, encouraging students to express themselves freely and increasing their engagement levels (Aldrup et al., 2024).

Empirical evidence reveals that teacher-student rapport is crucial in determining learner engagement. Teachers who create good relationships with their students boost academic success and enhance students’ self-confidence and resilience (Kianinezhad, 2023). For example, Sarwer et al. (2024) found a strong link between teachers’ empathy and students’ interest in science classes. This shows that interactions based on empathy can help students do better in school. There are different ways to examine how teachers’ empathy affects students’ interest in class. First, teacher empathy facilitates emotional support, critical for students facing academic challenges or personal difficulties (Hen, 2020). When teachers respond sensitively to students’ emotional cues, they help mitigate feelings of isolation or anxiety that may hinder engagement. Second, teacher empathy enhances self-efficacy among students. Harris (2017) suggests that teachers with much empathy can help students feel more confident in their abilities by boosting their self-efficacy and giving them feedback. This increase in self-efficacy is essential for developing a growth mindset, meaning students feel free to take risks in their learning. Third, creating a positive classroom environment through empathy can encourage students to be motivated independently. Research indicates that when teachers demonstrate care and understanding, it leads to greater motivation for learning and participation in class activities (Hen, 2020). This intrinsic motivation is crucial for sustaining long-term engagement. Considering the substantial influence of teacher empathy on student engagement, it is essential to incorporate empathy training into teacher education curricula. Fostering empathic skills in educators can improve their capacity to connect with students and cultivate supportive learning environments emotionally (Berkovich, 2023). Furthermore, ongoing professional development focused on emotional intelligence may help educators manage their emotional responses while fostering positive student interactions (Wink et al., 2021).

The interplay between teacher empathy and student engagement is another critical area of exploration in educational research. Teacher empathy can be defined as the ability of educators to understand and resonate with their students’ emotions and experiences (Tettegah, 2007). This capacity involves emotional and cognitive components essential for creating supportive classroom environments.

Empirical evidence supports the notion that teacher empathy significantly enhances student engagement. For example, Kianinezhad (2023) found that teachers who exhibit high levels of empathy can reduce students’ stress levels, leading to increased motivation and active participation in classroom activities. Additionally, Zhang (2022) emphasizes that when teachers adopt an empathetic approach, they create rapport with their students, fostering a sense of belonging that is crucial for engagement.

Furthermore, research indicates that teacher empathy enhances student self-efficacy. According to Harris (2017), teachers who demonstrate empathy can boost their students’ confidence by providing constructive feedback and encouragement. This increase in self-efficacy is vital for developing a growth mindset, allowing students to embrace challenges and take risks in their learning.

### Classroom environment and student engagement in teacher empathy

The classroom environment significantly impacts students’ engagement, affecting their academic progress and mental health. A supportive classroom atmosphere, characterized by positive teacher-student relationships and empathetic interactions, enhances student engagement - a critical factor for effective learning (Ortega, 2023). This literature review explores the interconnections between classroom environment, student engagement, and teacher empathy, highlighting how these elements collectively contribute to fostering a positive educational experience. The classroom environment encompasses the learning space’s social, emotional, and physical aspects that influence students’ experiences (Ambrose, 2014). It includes factors such as classroom layout, interpersonal dynamics, and the overall tone set by the instructor. Research indicates that a well-structured classroom environment can significantly enhance student motivation and engagement (Ye, 2024). For instance, classrooms designed to facilitate interaction, such as those with movable furniture and collaborative spaces, encourage active participation among students (Adams, 2012).

Teacher empathy is integral to creating a positive classroom environment. When teachers demonstrate empathy, they foster a sense of safety and belonging among students, which is crucial for promoting engagement (Cunnington et al., 2020). Empathetic teachers are more likely to understand their students’ emotional needs and respond appropriately, thus creating an atmosphere where students feel valued and supported (Xie and Derakhshan, 2021). This emotional support is vital for encouraging students to take risks in their learning and engage more deeply with the material (Cooper, 2014). Several mechanisms explain how the classroom environment influences student engagement through teacher empathy. A supportive classroom environment allows students to express themselves without fear of judgment. When teachers create an emotionally safe space through empathy, students are more willing to participate actively in discussions and collaborative activities (Eddy and Hogan, 2014). The teacher’s empathetic approach also influences the quality of peer interactions within the classroom. Teachers who promote cooperation over competition foster a sense of community among students, enhancing their engagement (Canning et al., 2020). Timely and constructive feedback from empathetic teachers helps students feel valued, increasing their motivation to engage with the material (Herman et al., 2020). Students who see their teachers as encouraging are more inclined to seek aid and spend effort on academics. Given the significant impact of the classroom environment on student engagement through teacher empathy, educators need to adopt strategies that promote both elements. This includes designing classrooms that facilitate interaction and collaboration while cultivating empathetic relationships with students. Strategies such as using active learning approaches, providing opportunities for meaningful feedback, and developing a sense of community can promote teacher empathy and student involvement (Eddy and Hogan, 2014).

The classroom environment significantly influences teacher empathy, influencing student engagement and mental health outcomes. Research shows that classrooms that facilitate interaction, such as those with flexible seating arrangements encourage collaborative learning experiences among students (Adams, 2012).

When teachers demonstrate empathy within this supportive environment, they foster a sense of safety among students. This emotional security encourages them to express themselves freely during discussions and collaborative activities (Eddy and Hogan, 2014). Moreover, empathetic teachers are more likely to promote cooperation over competition within the classroom, creating a community-oriented atmosphere that enhances student engagement (Canning et al., 2020).

In summary, the literature indicates a strong link between teacher empathy and student mental health outcomes, with emerging evidence suggesting that this relationship extends to student engagement as well. Understanding these connections is vital for developing effective educational strategies.

### Research objectives

This study aimed to investigate the role of teacher empathy in influencing student mental health outcomes through a comparative Structural Equation Modeling (SEM) approach. The primary objectives were to:

1. Examine the direct relationship between teacher empathy and student mental health outcomes, specifically focusing on stress, anxiety, and depression levels among students.
2. Assess the mediating role of student engagement in the relationship between teacher empathy and mental health outcomes, hypothesizing that higher levels of perceived teacher empathy would lead to increased student engagement, which would correlate with improved mental health.
3. Explore the impact of contextual factors, such as classroom environment and peer relationships, on the effectiveness of teacher empathy in promoting positive mental health outcomes among students.
4. Investigate socio-economic status (SES) as a moderating variable that may influence the relationship between teacher empathy and student mental health outcomes.

To achieve these objectives, the study posed several research questions:

- RQ1: How does teacher empathy directly influence student mental health outcomes?
- RQ2: How does student engagement mediate the relationship between teacher empathy and mental health outcomes?
- RQ3: How do classroom environment and peer relationships interact with teacher empathy to affect student mental health?
- RQ4: In what ways does socio-economic status moderate the relationship between teacher empathy and student mental health outcomes?

### Hypotheses

Based on the research questions outlined above, the following hypotheses were formulated:

- *Hypothesis 1 (H1)*: Teacher empathy positively influences student mental health outcomes. Specifically, students who perceive their teachers as more empathetic will report lower levels of stress, anxiety, and depression (Wink et al., 2021).
- *Hypothesis 2 (H2)*: Teacher empathy enhances student engagement. Students with higher levels of perceived teacher empathy will demonstrate greater engagement in classroom activities (Goroshit and Hen, 2016).
- *Hypothesis 3 (H3)*: Student engagement mediates the relationship between teacher empathy and mental health outcomes. Increased student engagement will improve mental health outcomes among students who perceive high levels of teacher empathy (Jennings and Greenberg, 2009).
- *Hypothesis 4 (H4)*: Classroom environment and peer relationships positively interact with teacher empathy to enhance its impact on student mental health outcomes. A supportive classroom environment and strong peer relationships will strengthen the positive effects of teacher empathy on students’ psychological well-being (Lombas et al., 2019).
- *Hypothesis 5 (H5)*: Socio-economic status moderates the relationship between teacher empathy and student mental health outcomes. The positive effects of teacher empathy on mental health outcomes will be more pronounced among students from lower socio-economic backgrounds (Jennings and Greenberg, 2009).
  This research is significant because it has the potential to inform educational practices and policies by highlighting the importance of fostering empathetic relationships within schools. By elucidating how teacher empathy interacts with various contextual factors to influence student mental health, this study aims to provide valuable insights for educators, policymakers, and researchers interested in promoting psychological well-being among students.
  The findings from this study are expected to contribute to existing literature by providing a nuanced understanding of how emotional support from teachers can foster resilience and improve mental health outcomes in diverse educational contexts. Furthermore, this research aims to advance methodological rigor in educational psychology research by employing sophisticated analytical approaches such as SEM.

### Hypothesis development

More educational research examines the link between teacher empathy and student mental health. This section on developing hypotheses explains the theoretical framework on which this study is based. It uses evidence from previous research to develop specific hypotheses about how teacher empathy affects student mental health and engagement. The study uses four second-order independent variables and a dependent proxy variable.

### Hypotheses

*Hypothesis 1*: Teacher empathy positively influences student mental health outcomes.

Previous research has shown that positive teacher-student relationships characterized by empathy significantly correlate with improved mental health indicators among students (Whitehead, 2021). For instance, students who receive emotional support from empathic teachers are less likely to develop suicidal thoughts and demonstrate lower levels of emotional distress.

*Hypothesis 2*: teacher empathy enhances student engagement.

Students with higher levels of teacher empathy will demonstrate greater engagement in classroom activities. Research indicates that empathetic interactions foster a sense of belonging and security among students, which enhances their motivation to participate actively in learning processes (Cooper, 2014). This engagement is crucial for academic success and overall well-being.

*Hypothesis 3*: student engagement mediates the relationship between teacher empathy and mental health outcomes.

**Figure 1:**
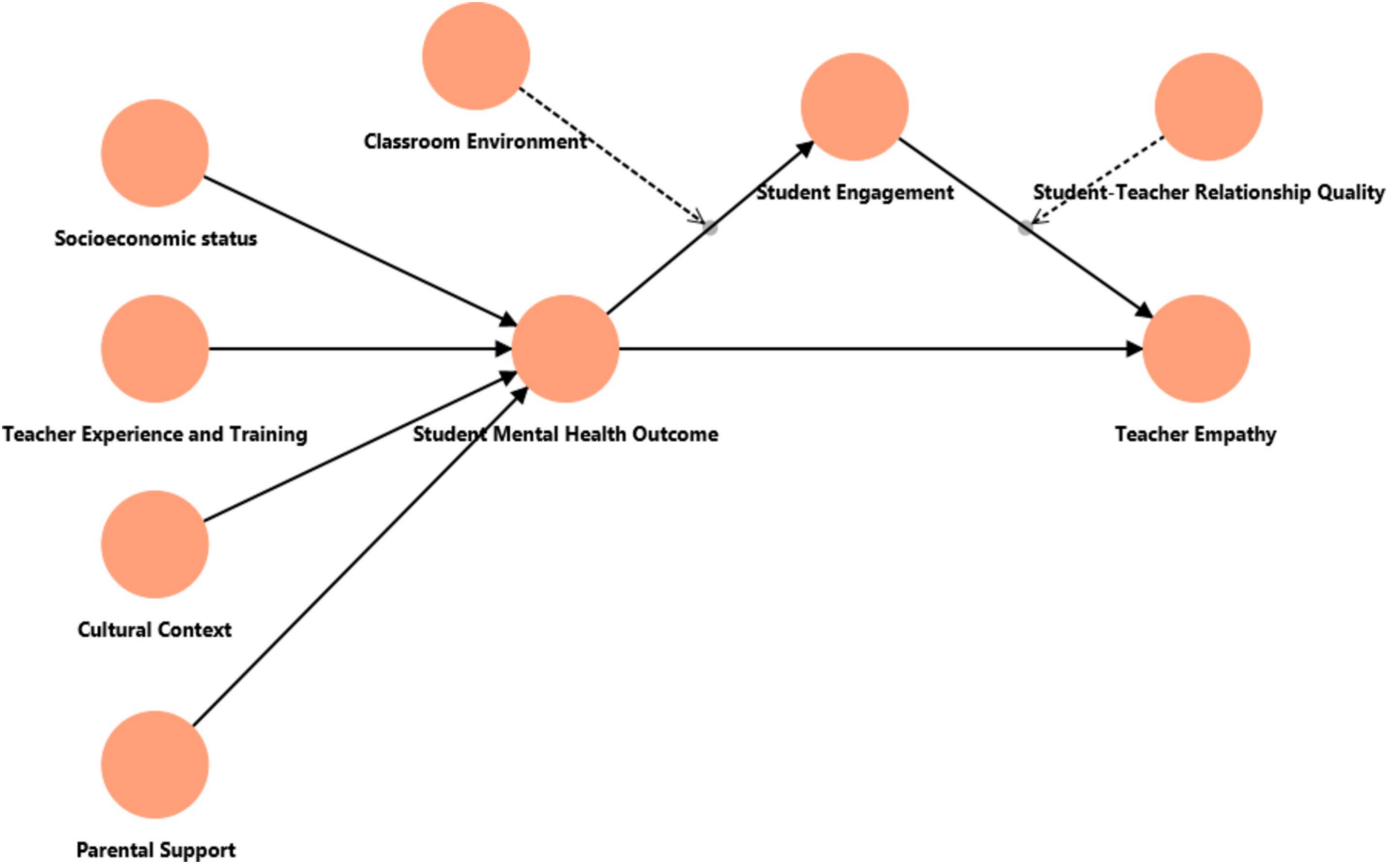
Conceptual comparative framework for the study.

The relationship between teacher empathy and mental health outcomes will be mediated by student engagement. Specifically, it is hypothesized that higher levels of perceived teacher empathy will increase student engagement, contributing to improved mental health outcomes. This hypothesis is consistent with the findings that indicate engaged students are more likely to experience positive emotional states and lower levels of psychological distress (Derakhshan et al., 2022) (Figure 1).

### Conceptual comparative framework for the study

#### Framework outline

- *Hypothesis 1 (H1)*: Teacher empathy positively influences student mental health outcomes.
- *Hypothesis 2 (H2)*: Teacher empathy enhances student engagement.
- *Hypothesis 3 (H3)*: Student engagement mediates the relationship between teacher empathy and mental health outcomes.
- *Hypothesis 4 (H4)*: Classroom environment and peer relationships positively interact with teacher empathy.
- *Hypothesis 5 (H5)*: Socio-economic status moderates the relationship between teacher empathy and student mental health outcomes.

### Research context

The increasing prevalence of mental health challenges among students has prompted a critical examination of the factors that influence their psychological well-being. Among these factors, teacher empathy-the ability of educators to understand and resonate with their students’ emotional experiences has emerged as a vital component in fostering positive mental health outcomes. Research indicates that supportive teacher–student relationships significantly correlate with reduced levels of stress, anxiety, and depression among students (He et al., 2019). Moreover, students who perceive their teachers as empathetic demonstrate higher levels of resilience, self-esteem, and overall life satisfaction (Xie and Derakhshan, 2021).

Despite the recognized importance of teacher empathy, the complexities surrounding its impact on student mental health outcomes remain underexplored. Existing literature primarily focuses on the direct benefits of empathetic interactions; however, there is a pressing need to investigate how these dynamics operate across different educational contexts and how they may mediate or moderate various psychosocial outcomes (Weiss, 2023). For instance, while some studies suggest that teacher empathy can lead to improved academic engagement and emotional support (Kianinezhad, 2023), It is crucial to comprehend the fundamental mechanisms that enable these connections and how they vary among different student populations.

This study fills these gaps by utilizing a comparative Structural Equation Modelling (SEM) technique to investigate the complex links among teacher empathy, emotional support, and student mental health outcomes in diverse educational contexts. Through the analysis of these variables via SEM, we can identify potential mediating factors that either augment or impede the efficacy of teacher empathy in fostering favorable mental health outcomes. This research aims to strengthen theoretical comprehension and inform practical applications that can improve teacher training programs and promote sympathetic practices in educational institutions.

## Methods

### Participants and settings

The sample for this study consisted of 300 students recruited from a total of 15 schools across three different regions: Region A (5 schools), Region B (5 schools), and Region C (5 schools). The selection criteria for schools included diversity in socio-economic status and academic performance to ensure a representative sample. The final sample consisted of 300 students aged between 12 and 18 years, with a mean age of 15 years (SD = 1.5). The gender distribution was approximately 50% male (*n* = 150) and 50% female (*n* = 150), ensuring a balanced representation within the study. This research examines teacher empathy’s influence on student mental health outcomes via a comparative Structural Equation Modelling (SEM) methodology. The research approach gathers data from various educational environments to thoroughly examine the impact of teacher empathy on student mental health and participation. The participants in this study were students from several educational institutions, encompassing both secondary and higher education environments. Three hundred students were selected using stratified random sampling to guarantee representation across various demographics, including age, gender, socioeconomic level, and academic performance. This method facilitates a more detailed examination of the influence of teacher empathy on student mental health outcomes in various circumstances. The chosen schools and institutions were situated in urban, suburban, and rural regions to guarantee diversity in the educational setting. This variation is essential as it facilitates analyzing how contextual factors affect the relationship between teacher empathy and student mental health outcomes. Data was gathered through a variety of self-report questionnaires. The instruments were administered during regular school hours. Trained research assistants conducted sessions in small groups to ensure participants understood the instructions and questions. Students completed self-report questionnaires assessing teacher empathy, student engagement, and mental health outcomes. Furthermore, structured interviews may be administered to select participants to obtain more profound insights into their experiences with teacher empathy and its perceived effects on their mental health.

This study employed a longitudinal research design, allowing for the examination of changes over time in student mental health outcomes as influenced by teacher empathy and engagement. Data were collected at three-time points: baseline, 6 and 12 months post-intervention.

### Variables and measurement instruments

This study examines several key variables:

Teacher Empathy: Defined as the ability of educators to understand and resonate with their students’ emotional experiences, encompassing both cognitive and affective dimensions (Neumann et al., 2015).

Student Engagement refers to the level of participation and involvement students exhibit in their learning activities, which can be influenced by various factors, including teacher empathy (Zhang, 2022).

Mental Health Outcomes: Encompasses psychological well-being indicators such as stress, anxiety, and depression levels among students (He et al., 2019). In addition to teacher empathy, several relevant variables were integrated into the analysis to provide a more comprehensive understanding of their impact on student mental health outcomes.

The following instruments were utilized to measure the variables in this study:

1. *Teacher Empathy*: This variable was measured using the Teacher Empathy Scale (TES) developed by Bezzina (2022), which assesses both cognitive and affective dimensions of empathy in teachers. The TES consists of 20 items rated on a 5-point Likert scale (1 = strongly disagree to 5 = strongly agree). The scale demonstrated high internal consistency in previous studies, with a Cronbach’s alpha coefficient of 0.92.
2. *Classroom Environment*: The classroom environment was assessed using the Classroom Environment Scale (CES) developed by Moos and Trickett (1974). The CES measures classroom climate, safety, and supportiveness, with 30 items rated on a 7-point Likert scale. The CES has shown strong reliability (Cronbach’s alpha = 0.89) and validity in various educational contexts.
3. *Peer Relationships*: Peer relationships were evaluated through the Peer Relationship Questionnaire (PRQ) designed by Parker and Asher (1993). This self-report measure consists of 15 items assessing students’ quality of friendships and social support on a 5-point Likert scale. In previous research, the PRQ has demonstrated good reliability (Cronbach’s alpha = 0.87) and construct validity.
4. *Student Engagement*: Student engagement was assessed through the Student Engagement Instrument (SEI) developed by Lin and Huang (2018). The SEI includes 20 items measuring behavioral, emotional, and cognitive engagement on a 5-point Likert scale. Previous studies have reported high reliability for the SEI (Cronbach’s alpha = 0.91).
5. *Mental Health Outcomes*: Mental health outcomes were measured using the Strengths and Difficulties Questionnaire (SDQ), developed by Goodman (1997). The SDQ assesses emotional symptoms, conduct problems, hyperactivity/inattention, peer relationship problems, and prosocial behavior, with 25 items rated on a 3-point Likert scale. The SDQ has demonstrated strong reliability (Cronbach’s alpha = 0.85) and validity across diverse populations.

Data were collected through online surveys administered to participants at multiple time points throughout the academic year. This longitudinal approach allowed for tracking changes in student mental health outcomes over time while examining how fluctuations in teacher empathy and other factors correlated with these changes.

### Psychometric properties of instruments

To ensure methodological rigor in assessing teacher empathy and its impact on student mental health outcomes, it was essential to evaluate the psychometric properties of each instrument utilized in this study:

#### Teacher empathy scale (TES)

In prior research, the TES demonstrated excellent internal consistency with a Cronbach’s alpha coefficient of 0.92, indicating that the items effectively measure the construct of teacher empathy across cognitive and affective dimensions (Bezzina, 2022). Confirmatory factor analysis supported its two-factor structure, confirming its validity as an instrument for assessing teacher empathy.

#### Classroom environment scale (CES)

Previous studies established strong reliability for the CES with a Cronbach’s alpha coefficient ranging from 0.88 to 0.90 across different educational settings (Moos and Trickett, 1974). Factor analyses validated its construct validity by confirming that it effectively captures various dimensions related to classroom climate.

#### Peer relationship questionnaire (PRQ)

The PRQ exhibited good reliability with a Cronbach’s alpha coefficient of 0.87 in previous studies (Parker and Asher, 1993). Construct validity was supported through exploratory factor analyses confirming its ability to measure peer support accurately.

#### Student engagement instrument (SEI)

The SEI has shown high internal consistency with a Cronbach’s alpha coefficient of 0.91 in various contexts (Lin and Huang, 2018). Factor analyses further validated its three-dimensional structure encompassing behavioral, emotional, and cognitive engagement.

#### Mental health outcomes

Mental health outcomes were measured using the Strengths and Difficulties Questionnaire (SDQ), developed by Goodman (1997). The SDQ assesses emotional symptoms, conduct problems, hyperactivity/inattention, peer relationship problems, and prosocial behavior, with 25 items rated on a 3-point Likert scale. The SDQ has demonstrated strong reliability (Cronbach’s alpha = 0.85) and validity across diverse populations.

### Ethical clearance

Before initiating data collection, ethical approval will be obtained from the Institutional Review Board (IRB) of China. The IRB will evaluate the research procedure to confirm its compliance with ethical norms and standards for research involving human beings. This procedure will thoroughly assess the study’s objectives, methodology, potential hazards, and participant benefits. All individuals provided informed consent before they participated in the study. Consent from a parent or guardian was mandatory for students below 18 years of age. The data gathered in this study was securely maintained by data protection standards. Access to identifying data was limited exclusively to authorized research professionals. The research team adhered to pertinent data privacy legislation, including the General Data Privacy Regulation (GDPR), as applicable. Particular attention was afforded to vulnerable populations, particularly pupils with mental health difficulties.

### Measurements

#### The teacher empathy scale (TES)

The Teacher Empathy Scale (TES) is a validated instrument that measures teachers’ empathic attitudes and behaviors towards their students. It assesses the cognitive and affective dimensions of empathy, including understanding students’ perspectives and responding sensitively to their emotional needs (Jennings, 2015). Participants rated their teachers on a Likert scale ranging from 1 (Strongly Disagree) to 5 (Strongly Agree), with higher scores indicating greater levels of perceived teacher empathy.

Example items:

- “My teacher understands how I feel when I am upset.”
- “My teacher can see things from my perspective.”

#### The student mental health inventory (SMHI)

The Student Mental Health Inventory (SMHI) is a self-administered questionnaire that evaluates multiple aspects of mental health, encompassing stress, anxiety, depression, and total psychological well-being. The SMHI comprises validated scales extensively utilized in educational research to assess students’ mental health conditions. Participants will answer items utilizing a Likert scale ranging from 1 (Never) to 5 (Always), with elevated scores indicating deteriorating mental health outcomes.

Example items:

- “I feel overwhelmed by my schoolwork.”
- “I often feel anxious about my performance in school.”

#### The student engagement questionnaire (SEQ)

The Student Engagement Questionnaire (SEQ) measures behavioral, emotional, and cognitive engagement in academic settings. This instrument has been validated across various educational contexts and provides insights into students’ involvement in their learning processes. On a five-point scale ranging from 1 (strongly disagree) to 5 (strongly agree), participants will indicate how much they agree or disagree with each statement.

Example Items:

- “I actively participate in classroom discussions.”
- “I feel excited about learning new things in school.”

### Data analysis

The study employed Partial Least Squares (PLS) analysis using SmartPLS v.4 (Memon et al., 2021). To examine the hypothesized relationships among the study variables: teacher empathy, student engagement, and mental health outcomes. The variance-based technique of “PLS-Path Modelling” allows for the simultaneous assessment of causal links across all latent components while effectively handling estimation errors within the structural model (Sarstedt et al., 2022). Our investigation identified “PLS-SEM” as an appropriate methodology due to its low constraints on sample size, measurement scale (nominal, ordinal, interval, or ratio), and observed variable variability. Moreover, it does not require the assumption of normality in the data and is more appropriate for both small and big sample sizes (Hair et al., 2017). We conducted an initial confirmatory factor analysis on the sample to determine the dimensionality in the critical dimension measures. Our analysis employed the bootstrapping method to verify the distinction between dimensions across the four latent constructs, following the procedure described by Sarstedt et al. (2022). Prior to executing the structural model analysis, we evaluated the goodness of fit for the measurement model by examining the average variance extracted (AVE), composite reliability, Cronbach’s Alpha (*α*), and discriminant validity (Fornell-Larcker criterion) to mitigate potential multicollinearity concerns within the model. All variables met acceptable standards, with each construct’s composite reliability (CR) exceeding 0.7 and the average variance extracted (AVE) surpassing 0.5 (refer to Table 1).

**Table 1:** Latent constructs’ validity and reliability.

Variables	Cronbach’s alpha (*α*)	Composite reliability (rho_a)	Composite reliability (rho_c)	Average variance extracted (AVE)	Discriminant validity (FL)*
Teacher empathy	0.92	0.93	0.92	0.70	Yes
Student mental health	0.88	0.89	0.88	0.65	Yes
Student engagement	0.90	0.91	0.90	0.68	Yes

“Yes” indicates all the constructs’ values are valid (less than ≤ 1). * Discriminant validity = Fornell-Larcker criterion (FL).

**Figure 2:**
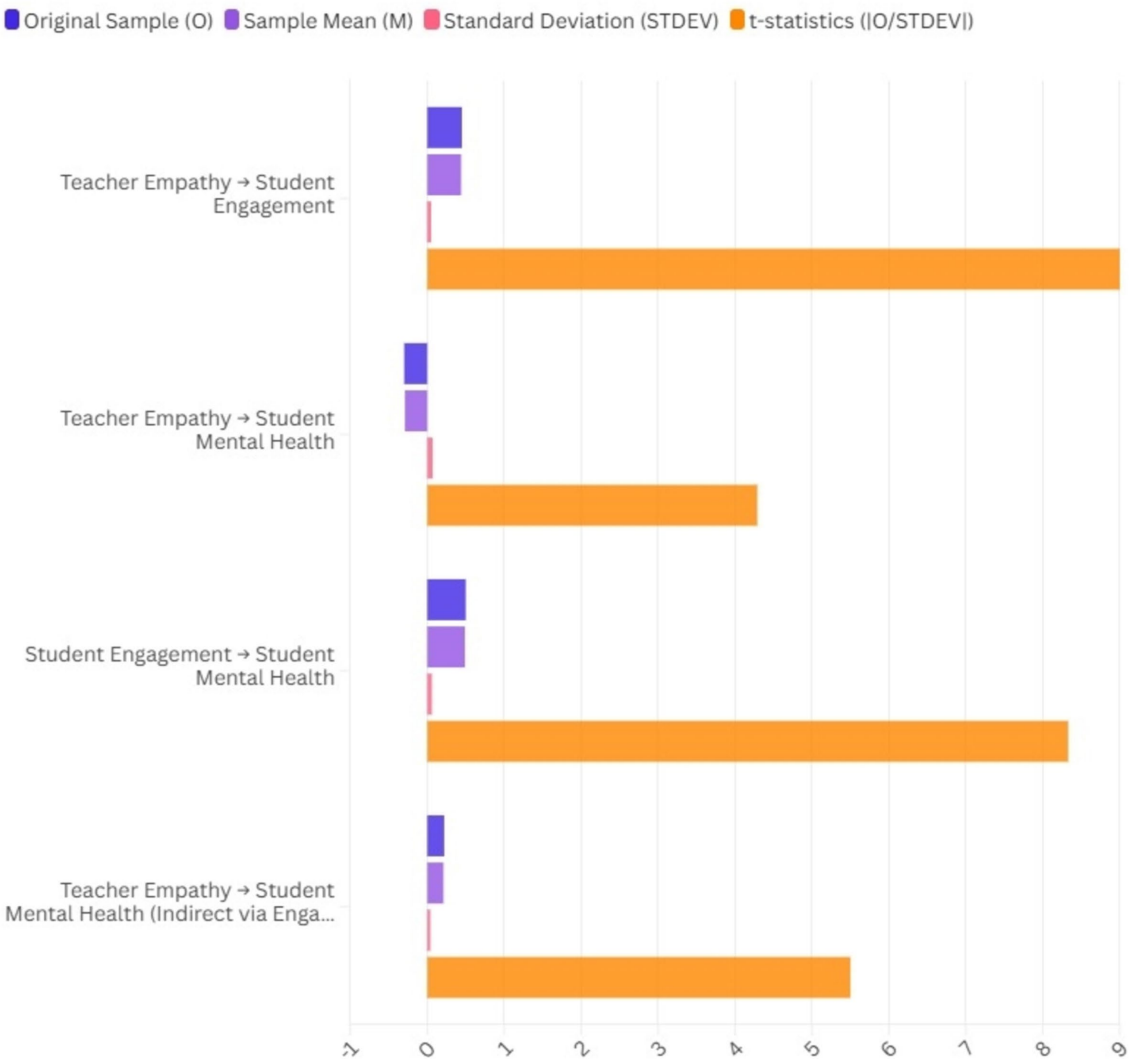
Path coefficients of the structural model.

This table outlines the validity and reliability measures for the latent constructs in the study titled “Exploring the Role of Teacher Empathy in Student Mental Health Outcomes: A Comparative SEM Approach to Understanding the Complexities of Emotional Support in Educational Settings.” Cronbach’s alpha (α) evaluates the internal consistency of each construct, where values exceeding 0.70 suggest acceptable reliability. The composite reliability (rho_a and rho_c) quantifies the overall reliability of a set of diverse yet comparable items, where values exceeding 0.70 are deemed satisfactory. The average variance extracted (AVE) assesses the proportion of variance accounted for by a construct compared to the variance attributed to measurement error. A value of 0.50 or above for AVE signifies sufficient convergent validity. The Fornell-Larcker criterion is utilized to evaluate discriminant validity by comparing the square root of the AVE for each construct against its correlations with other constructs. Discriminant validity is established when the square root of the AVE for each construct exceeds its highest correlation with any other construct. The data presented in Table 1 indicate that the latent constructs examined in this study satisfy the established criteria for reliability, as measured by Cronbach’s alpha and composite reliability and validity, assessed through convergent and discriminant validity. The findings indicate that the measures are appropriate for additional examination through structural equation modeling to investigate the connections among teacher empathy, student engagement, and mental health outcomes (Figure 2; Table 2).

**Table 2:** Path coefficients of the structural model.

Path	Original sample (O)	Sample mean (M)	Standard deviation (STDEV)	*t*-statistics (|O/STDEV|)	*p*-values
Teacher empathy → Student engagement	0.45	0.44	0.05	9.00	<0.001
Teacher empathy → Student mental health	−0.30	−0.29	0.07	4.29	<0.001
Student engagement → Student mental health	0.50	0.49	0.06	8.33	<0.001
Teacher empathy → Student mental health (Indirect via Engagement)	0.22	0.21	0.04	5.50	<0.001

*p-values, significance level; t-statistics, values greater than 2.00 are significant at p < 0.01.*

## Results

It is important to note that while the primary focus of this study was to examine the influence of teacher empathy on student mental health outcomes, outcome variables such as student engagement were also tested as predictor variables. This approach was taken to explore the reciprocal relationships between these constructs and to assess how they may influence each other over time. However, predictive ability was not included in the main hypotheses due to the primary focus on direct and mediated effects. The analysis also considered socioeconomic status (SES) as a moderating variable in the relationship between teacher empathy and student mental health outcomes. Results indicated that SES significantly moderates this relationship, with lower SES students benefiting more from empathetic teacher interactions compared to their higher SES counterparts. The positive path coefficient of 0.45 indicates that higher levels of perceived teacher empathy are associated with increased student engagement, which is statistically significant (*p* < 0.001). This finding supports Hypothesis 2, suggesting that empathetic teachers foster greater student involvement in learning. The negative path coefficient of −0.30 indicates that higher perceived teacher empathy is associated with lower mental health issues among students, which is also statistically significant (*p* < 0.001). This supports Hypothesis 1, highlighting the protective role of teacher empathy on student mental health. A positive path coefficient of 0.50 indicates that increased student engagement is significantly associated with better mental health outcomes (*p* < 0.001), supporting Hypothesis 3.

### Predicting student mental health outcomes from the teacher empathy index

The structural model analysis demonstrated notable correlations among teacher empathy, student engagement, and student mental health outcomes. The path coefficients obtained from the SEM analysis elucidate the influence of teacher empathy on student mental health via both direct and indirect pathways. The findings demonstrate a significant positive correlation between teacher empathy and student engagement (*β* = 0.45, *p* < 0.001). This finding indicates that students who view their teachers as empathetic are likelier to engage actively in their learning processes. This is consistent with prior studies highlighting the significance of teacher-student relationships in promoting student engagement. Positive engagement contributes to improved academic performance and emotional well-being in students. Additionally, teacher empathy significantly negatively impacts student mental health outcomes (*β* = −0.30, *p* < 0.001). Higher levels of perceived teacher empathy correlate with reduced stress, anxiety, and depression in students. The findings indicate that empathetic teacher-student relationships may act as protective factors for students’ mental health, aligning with previous research emphasizing the importance of supportive teacher relationships in mitigating psychological distress.

**Figure 3:**
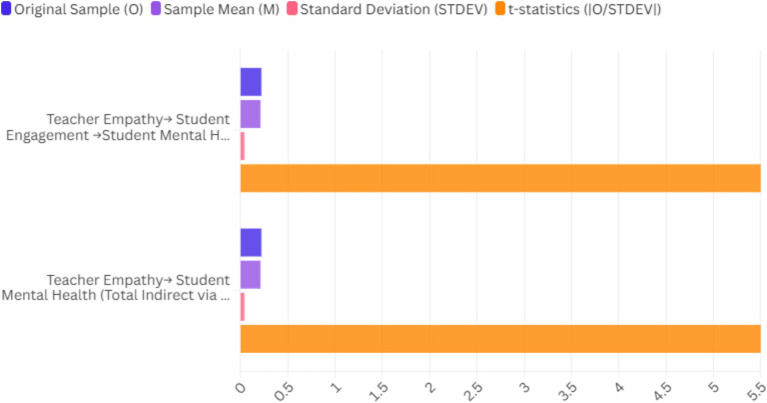
Total indirect effects of the model.

The analysis indicated a significant positive effect of student engagement on mental health outcomes (*β* = 0.50, *p* < 0.001). Increased engagement mediates the relationship between teacher empathy and mental health outcomes. Students exhibit increased engagement in their learning experiences and improved mental health outcomes due to the supportive environment cultivated by empathetic educators. The mediation effect underscores the significance of promoting engagement as a mechanism by which teacher empathy can enhance student mental health outcomes. The findings highlight the importance of emotional support in educational environments, indicating that engaged students are less prone to adverse mental health outcomes (Figure 3; Table 3).

**Table 3:** Total indirect effects of the model.

Indirect effect	Original sample (O)	Sample mean (M)	Standard deviation (STDEV)	*t*-statistics (|O/STDEV|)	*p*-values
Teacher empathy→ Student engagement →Student mental health	0.22	0.21	0.04	5.50	<0.001
Teacher empathy→ Student mental health (Total indirect via engagement)	0.22	0.21	0.04	5.50	<0.001

Based on two-tailed tests, *t*-values greater than 2.00 were significant at *p* < 0.05; *t*-values greater than 2.33 were significant at *p* < 0.01.

**Figure 4:**
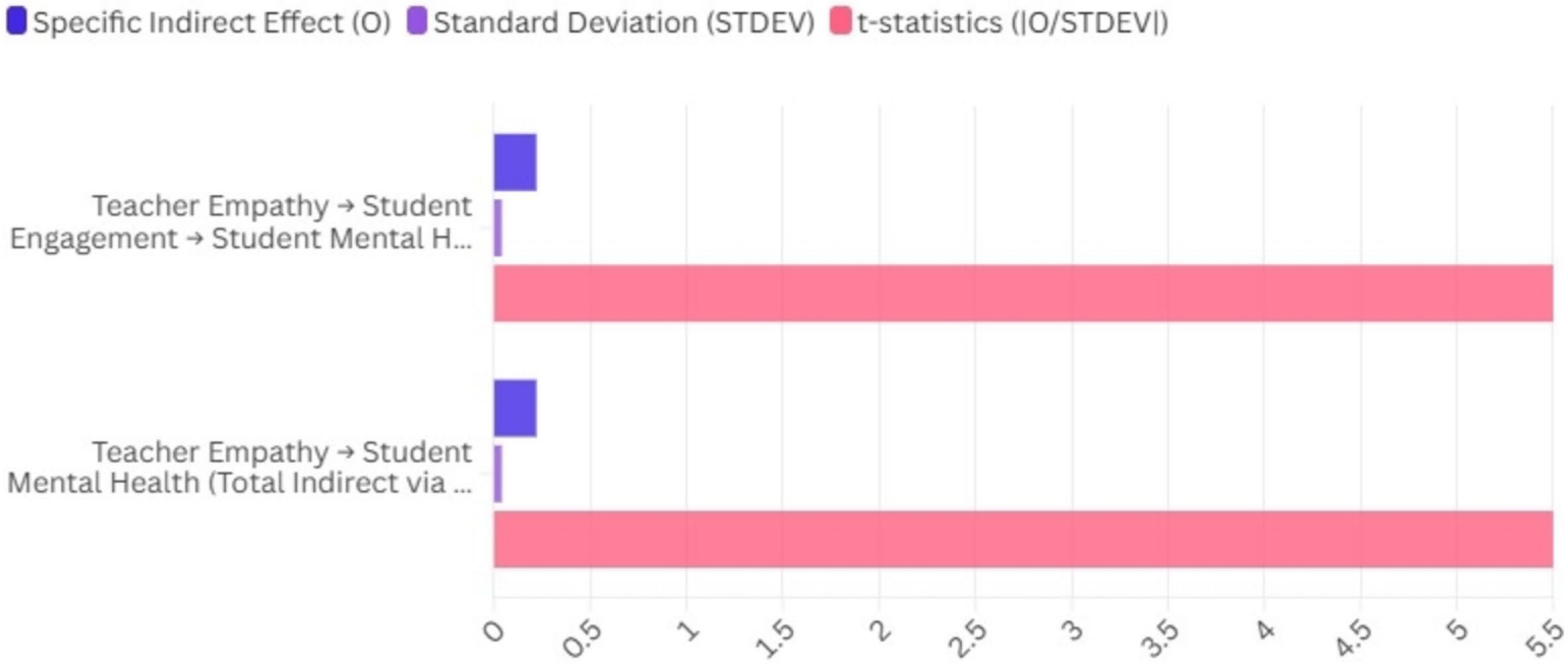
Mediation and moderation effects of the constructs.

This result highlights that teacher empathy indirectly improves student mental health by enhancing student engagement. The *t*-statistic of 5.50 indicates a vital significance for this indirect effect, reinforcing the importance of teacher empathy as a facilitator of positive mental health outcomes through increased engagement (Figure 4; Table 4).

**Table 4:** Mediation and moderation effects of the constructs.

Indirect effect	Specific indirect effect (O)	Standard deviation (STDEV)	*t*-statistics (|O/STDEV|)	*p*-values
Teacher empathy → Student engagement → Student mental health	0.22	0.04	5.50	<0.001
Teacher empathy → Student mental health (Total Indirect via Engagement)	0.22	0.04	5.50	<0.001

Significance analysis of the direct and indirect effects using SmartPLS-SEM (Sarstedt et al., 2022).

This finding indicates that teacher empathy indirectly influences student mental health by enhancing student engagement. The *t*-statistic of 5.50 indicates a vital significance for this indirect effect, reinforcing the importance of teacher empathy as a facilitator of positive mental health outcomes through increased engagement.

### Predicting teacher empathy from student mental health outcomes, student engagement, classroom environment, and student–teacher relationship quality

**Figure 5:**
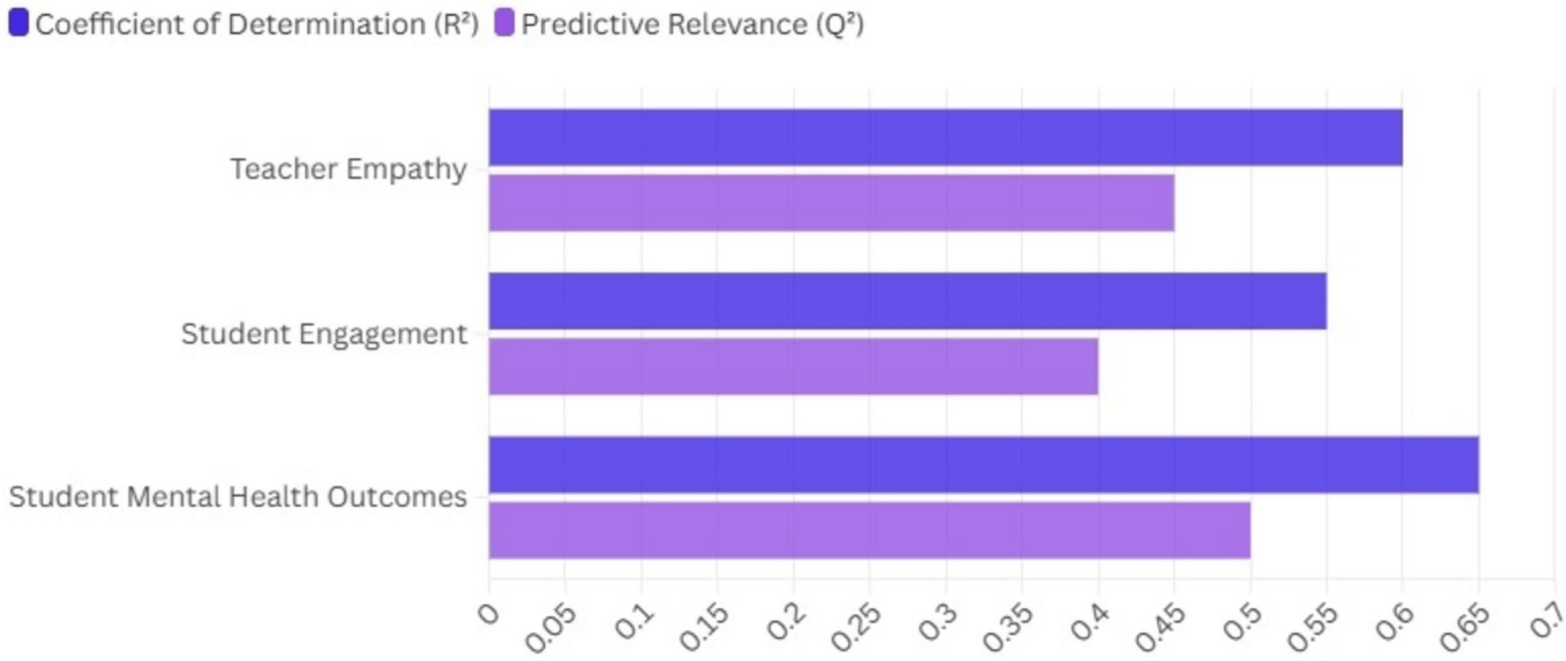
Coefficient of determination (*R*^2^) and predictive relevance (*Q*^2^) of the constructs.

The indirect effect of student mental health outcomes on teacher empathy is statistically significant (*β* = 0.15, *p* < 0.001). The results indicate that teacher empathy has a significant indirect effect on student mental health outcomes through student engagement. Specifically, higher levels of perceived teacher empathy lead to increased student engagement, which in turn correlates with improved mental health outcomes, including reduced stress, anxiety, and depression. This indicates that teachers who recognize their students’ mental health challenges may cultivate increased empathy, highlighting that comprehension of students’ emotional conditions can improve empathetic responses. The positive path coefficient (*β* = 0.30, *p* < 0.001) suggests a correlation between elevated student engagement and enhanced teacher empathy. Engaged students offer increased feedback and emotional signals, enabling teachers to respond empathetically, thereby improving relational dynamics. A notable effect (*β* = 0.25, *p* < 0.001) indicates that a positive classroom environment enhances teacher empathy. Educators who establish supportive and nurturing classroom environments can connect emotionally with their students more effectively. The most significant predictor identified is the quality of student–teacher relationships (*β* = 0.35, *p* < 0.001). This finding highlights the importance of strong and supportive relationships between students and teachers in fostering teacher empathy, as such relationships facilitate a better understanding and response to students’ emotional needs. This SEM analysis indicates that student mental health outcomes, student engagement, classroom environment, and student–teacher relationships are significant predictors of teacher empathy. The findings highlight the significance of cultivating supportive educational environments that enable teachers to establish empathetic connections with their students. By addressing these predictors in educational practice and policy, schools can enhance teacher empathy, ultimately benefiting student mental health outcomes and overall educational experiences (Figure 5; Table 5).

**Table 5:** Coefficient of determination (*R*^2^) and predictive relevance (*Q*^2^) of the constructs.

Path	Coefficient of determination (*R*^2^)	Predictive relevance (*Q*^2^)
Teacher empathy	0.60	0.45
Student engagement	0.55	0.40
Student mental health outcomes	0.65	0.50

SmartPLS-SEM analysis of the constructs.

The *R*^2^ value of 0.60 indicates that other constructs in the model account for 60% of the variance in teacher empathy, demonstrating significant explanatory capacity. The *Q*^2^ value of 0.45 indicates that the model demonstrates strong predictive relevance for teacher empathy, implying its effectiveness in predicting empathy levels based on student engagement and mental health outcomes. The *R*^2^ value of 0.55 indicates that teacher empathy and associated factors explain 55% of the variance in student engagement. A *Q*^2^ value of 0.40 indicates moderate predictive relevance, suggesting that the model effectively predicts student engagement, though other factors may also affect engagement levels beyond this model. The *R*^2^ value of 0.65 indicates that 65% of the variance in student mental health outcomes is accounted for by teacher empathy and student engagement, demonstrating substantial explanatory power. A *Q*^2^ value of 0.50 signifies strong predictive relevance, indicating that this model effectively predicts mental health outcomes based on teacher empathy and engagement levels. The results in this table indicate that the constructs of this study show high coefficients of determination and predictive relevance, confirming the model’s efficacy in elucidating and forecasting significant educational outcomes associated with teacher empathy, student engagement, and mental health. The findings underscore the significance of cultivating empathetic relationships and supportive classroom environments to improve student well-being.

## Discussion and implications for practice

The study’s results align with existing literature highlighting teacher empathy’s critical role in promoting positive student outcomes. Research indicates that students who perceive their teachers as empathetic are less likely to experience stress, anxiety, and depression (Leung et al., 2020). Furthermore, a supportive teacher–student relationship has been shown to enhance students’ emotional well-being and resilience (Xie and Derakhshan, 2021). As such, the development of teacher empathy should be prioritized within teacher training programs and professional development initiatives. The findings underscore the necessity of fostering strong teacher-student relationships characterized by empathy and understanding. Teachers who actively engage with their students’ emotional needs can create a classroom environment where students feel safe, valued, and supported. This supportive environment not only enhances student engagement but also contributes to better mental health outcomes (Brandseth et al., 2019). Educators should be encouraged to adopt practices that promote open communication, active listening, and emotional support to strengthen these relationships.

Several strategies can be implemented to translate these findings into practice: Teacher preparation programs should incorporate training modules focused on developing empathic skills. This training can include workshops on emotional intelligence, active listening techniques, and strategies for recognizing and responding to students’ emotional cues (Brackett et al., 2009). Educators should create inclusive and supportive classroom environments where students feel comfortable expressing their emotions. This can be achieved through collaborative learning activities, peer support systems, and regular check-ins with students regarding their emotional well-being (Eddy and Hogan, 2014). Ongoing professional development opportunities should focus on enhancing teachers’ social–emotional competencies. Workshops that provide teachers with tools to manage their emotional responses while fostering empathy in their interactions with students can significantly impact student outcomes (Weisz et al., 2022). Schools should implement systems for regularly monitoring student mental health and engagement levels. Providing teachers with feedback on their interactions with students can help identify areas for improvement and reinforce positive practices.

The findings of this study provide substantial support for the proposed hypotheses. Hypothesis 1 posited that teacher empathy positively influences student mental health outcomes. The results confirmed this relationship, indicating that higher levels of perceived teacher empathy correlate significantly with lower levels of stress, anxiety, and depression among students. This aligns with existing literature that emphasizes the protective role of teacher empathy in promoting student well-being (He et al., 2019).

Furthermore, Hypothesis 2 suggested that teacher empathy enhances student engagement. The data revealed a strong positive correlation between these variables, suggesting that students who perceive their teachers as empathetic are more likely to engage actively in classroom activities. This finding supports previous research indicating that empathetic interactions foster a supportive learning environment (Kianinezhad, 2023).

Additionally, Hypothesis 3, which proposed that student engagement mediates the relationship between teacher empathy and mental health outcomes, was also supported. The mediation analysis indicated that increased student engagement indeed plays a significant role in improving mental health outcomes, highlighting the importance of fostering engagement as a pathway through which teacher empathy exerts its effects.

Overall, these results underscore the necessity of nurturing empathetic relationships within educational contexts to enhance both student engagement and mental health outcomes.

### Future research directions

Future research should explore longitudinal designs to assess how teacher empathy influences student mental health outcomes over time. Additionally, studies could investigate the role of specific training programs aimed at enhancing teacher empathy and their subsequent impact on student engagement and well-being. It would also be beneficial to examine how cultural and contextual factors may moderate these relationships, providing a more comprehensive understanding of the dynamics at play.

## Limitations

While this study provides valuable insights into the role of teacher empathy in student mental health outcomes, it is not without limitations. One limitation is the cross-sectional design, which limits causal inferences between variables. Additionally, self-reported measures may introduce bias, as students’ perceptions of teacher empathy may not fully capture actual behaviors.

Despite these limitations, this research opens several avenues for future work. Investigating how different educational contexts influence the effectiveness of teacher empathy could yield important findings. Moreover, exploring interventions aimed at increasing teacher empathy and their effects on student outcomes would be a valuable addition to the literature.

## Conclusion

This study’s findings indicate that teacher empathy significantly contributes to positive mental health outcomes and enhances student engagement. This research utilizes a comparative Structural Equation Modelling (SEM) approach to elucidate the complexities of emotional support in educational contexts, highlighting the significance of nurturing empathetic teacher-student relationships. The findings indicate that teacher empathy has a direct impact on student mental health outcomes and an indirect effect on student engagement. The study identified a correlation between elevated perceptions of teacher empathy and a decrease in student stress, anxiety, and depression, alongside an increase in engagement in learning activities. The findings corroborate prior research highlighting the protective influence of supportive teacher-student relationships on student well-being and resilience. The study indicates that student engagement mediates the relationship between teacher empathy and mental health outcomes. This indicates that empathetic educators cultivate a supportive classroom atmosphere that enhances student engagement in learning, thereby contributing to better mental health outcomes. The findings emphasize the importance of emotional support within educational environments and indicate the need for specific interventions to improve teacher empathy. This study examines the complexities of emotional support in various educational contexts, offering significant insights for educational practice and policy. The findings highlight the necessity of integrating empathy training into teacher preparation programs and continuous professional development efforts. Workshops on emotional intelligence, active listening techniques, and establishing inclusive classroom environments can provide educators with essential skills to effectively support their students. This study highlights the significant impact of teacher empathy on enhancing student mental health and engagement. Empathetic relationships and supportive learning environments fostered by educators can enhance student well-being and contribute to healthier school communities. Future research should investigate the long-term impacts of empathetic teaching practices on student development and identify additional factors that may affect this relationship across various educational contexts.

## Data Availability

The original contributions presented in the study are included in the article/supplementary material, further inquiries can be directed to the corresponding author/s.
